# Sometimes determination and compromise thwart success: lessons learned from an effort to study copying and pasting in the electronic medical record

**DOI:** 10.1007/s40037-018-0427-8

**Published:** 2018-04-23

**Authors:** Jane P. Gagliardi, Mariah J. Rudd

**Affiliations:** 1 Department of Psychiatry and Behavioral Sciences, Duke University School of Medicine, Durham, USA; 2 Education & Faculty Development, Office of Continuing Professional Development, Virginia Tech Carilion School of Medicine, Roanoke, VA USA

**Keywords:** Electronic medical record, Communication, Professionalism, Lessons learned

## The story

Copying/pasting, note-forwarding, and templating are common practices in the electronic medical record (EMR) even though early observations indicated a widespread belief that such behaviours would be rare [1]. Recent surveys indicate much broader acceptance and existence of copying/pasting, and other purportedly efficiency-producing behaviours than initially was predicted [2]. Medical educators and clinicians have raised concerns about the impact on notes, which may be rendered bloated or meaningless [3, 4], as well as the impact on the patient-physician relationship, the physician-learner relationship, and the ability of learners to interact with patients [3, 5, 6]. Learners (and providers) engaging in copying/pasting and other EMR-based efficiency measures may have less in-depth knowledge about their patients [7]. Whether there is any causation in this correlation is not well studied, but studies in other fields suggest that technological multitasking may overwhelm individuals’ ability to attend to detail [8].

Educational impact of the EMR, particularly that of interpersonal and communication skills, has been discussed by previous authors [9, 10]. Of concern to us is the possibility that critically important aspects of learning (including direct patient care, professionalism and accountability) and patient care are suffering as metrics and efficiency measures are increasingly prioritized [11].

In order to better characterize the impact of copying/pasting information in the EMR on patient care and learning, we endeavoured to study patient- and learner-relevant outcomes. The initial proposal was to randomize clinical general medicine service teams to one of two conditions: (1) able to copy/paste in the EMR and (2) unable to paste information in the EMR. By randomizing groups of learners (including junior as well as senior members of the healthcare team) into one of these conditions, we expected to be able to observe how cut and paste activities impacted patient care and provider/learner outcomes. Patient care outcomes would include hospital length of stay, seven-day readmission, and mortality. Provider/learner outcomes would include faculty and resident retention of information relevant to the patients on their services under these conditions.

In 2011, when we made the initial proposal, our institution had just begun the planning phases of implementation of a new EMR. Given the elaborate logistics for transitioning to this EMR while maintaining quality and efficient patient care, health system leaders deemed our planned randomization-based methodology incompatible with larger health system goals. In other words, we were not given permission to pilot the intervention, program the EMR to allow and not allow copy/paste function by the service team, and collect and analyze real patient outcomes. What we had at this point was a study design that we felt would enable us to test our hypothesis; what we did not have was a context in which to implement the study.

Undaunted, in 2014, we sought to create a pilot study to test our hypothesis that copying/pasting has a measurable impact on learning and patient care. Without the ability to use the EMR and active clinical services to answer our research questions, we realized that we needed to narrow the scope of the study, including the study population and the outcomes we would measure. We set about attempting to create a study that might address the impact of copy/paste behaviours on patient-relevant outcomes and retention of information by a narrower group of healthcare providers, that is, residents, evaluating patients for the first time. The study we designed had two components. First, we planned to survey learners in the graduate medical education (GME) programs regarding their current perception and patterns of EMR use. These survey results would provide data on GME trainees’ perceptions of the utility of the EMR, their self-reported use of templates and copying/pasting behaviours in the EMR, and their observations on drawbacks and benefits for the EMR. Second, we would utilize an observed structured clinical examination (OSCE) to assess the impact of copying/pasting on care providers’ recall of patient-specific information, ability to glean new information about patients, and general medical knowledge. For this second component, we had hoped to be able to use the existing EMR in ‘sandbox’ or ‘playground’ mode. Other studies have been able to simulate the EMR in learning or training environments, and so we planned to duplicate this approach. We planned to have participants complete a first-time evaluation of a standardized patient in the OSCE and then write a new patient evaluation in the EMR, under time pressure, which we believed would simulate circumstances that lead to copying/pasting and/or templating behaviours. We planned to permit templating and copying/pasting behaviours in half of the new patient evaluations and disallow them in the other half, then compare accuracy of documentation and recall. The study was designed in two parts for a couple of reasons. First, we thought it would be interesting to compare self-reported vs. actual behaviours in the EMR. Second, we used the survey as a recruiting tool: at the conclusion of the survey we included an option to volunteer for an upcoming OSCE designed to assess the impact of behaviours in the EMR.

We received institutional funding to complete the survey and to cover costs associated with running the OSCE exercise. We then moved forward with developing and disseminating the baseline survey.

When it came time to assess the actual impact of copying/pasting and templating behaviours in the EMR, though, we again ran into trouble with our study design—this time because of insufficient financial support. The use of the existing EMR during the OSCE exercise was ‘approved’ by the institution, but funding was not awarded for the manipulation of the EMR (even in a ‘test’ mode) to meet our study design requirements. Although we had engaged in fact-finding and baseline research to ensure that we could use our EMR for an OSCE exercise, allowing for copying/pasting in some simulated clinical situations and not in others, we later learned that the logistics of incorporating the EMR in the OSCE environment would require purchasing eight computers or EMR-compatible tablets. We did not have funding to cover such costs. Furthermore, EMR technology support staff were busy addressing a multitude of other issues associated with the ongoing adaptation and implementation of the EMR in a large and diverse health system. In other words, institutional priorities did not include providing EMR technology support for the study. Even if we could identify funding, our institution would not be able to support the use of the EMR for this study. Given these institutional EMR restrictions, we found ourselves, again, with an idea for a study design to test our hypothesis but not a way to implement. How could we study the impact of copy/paste behaviours in the EMR with a study design that no longer had participants interacting with an EMR?

Determined to pursue our research interests, we began working on a compromise solution to our quandary. We needed a workaround that would mimic the EMR in a context where we could not use the EMR. After extensive discussion with our research team and research on resources within the institution, we decided that we could manipulate a survey software (already available on the computer workstations in the OSCE lab) to mimic the parts of the EMR that we needed. Thus, we could simulate a situation in which subjects would be ‘writing a new patient evaluation’ of the standardized patients in a situation in which they (1) could or (2) could not copy/paste available information into their evaluation. We had already written four cases for the OSCE, including information which was intentionally designed to contain some ‘pitfalls’ that might befall an individual undertaking automatic or copy/paste behaviour. These were also intended to be measurable in the quiz designed for administration post-OSCE. Perhaps we had found the solution to our research design challenges!

The OSCE was set up as a two-hour, four-station examination during which participants would have a total of 25 min for each case, with up to 15 min interacting with standardized patients and the remaining time dedicated to documenting the encounter in the ‘simulated EMR’ survey environment. In the simulated EMR environment, participants would see standardized patients in a randomized order, with the ability to copy/paste patient information in two of the four scenarios and not in the other two scenarios. Each of the scenarios had a ‘copy/paste-able’ survey and a ‘not copy/paste-able’ survey, so there were eight total possible conditions, of which each participant undertook four. At the conclusion of the two-hour OSCE, study subjects were asked to complete a 20-question quiz, which had five questions per station aimed at determining information subjects would have gathered from the encounters; the percent correct on each scenario’s questions was then to be correlated with the condition (copy-and-paste or no).

## Surprising outcomes

Survey results affirmed our suspicions that trainees were aware of potential pitfalls of copying/pasting but nevertheless engaged in the behaviour in their own use of the EMR. The number of trainees who reported *never* engaging in copy/paste activities in the EMR (37 of 137 respondents (27%)) was smaller than the number of trainees who admitted to copying/pasting information (100 of 137 respondents (73%)). Additionally, the majority of respondents reported frequently coming across notes in the EMR that had been copied/pasted. Qualitative survey results indicated trainees found copied documentation less than helpful in their evaluation of patients. This finding was not surprising but supported our impressions and hypothesis.

What was surprising (and disappointing) was the extent to which our OSCE-based study *did not* successfully capture the data we were hoping to collect. While we could incorporate a copy-and-paste (or not) function in the survey software, simulating the complex tasks involved in using an EMR was simply not possible using even cleverly-manipulated survey software. EMRs have complex interface designs in which users check multiple tabs, perform various activities, receive alerts, use templates, and interact with documents that often contain pre-populated information. The survey tool simply could not mimic this complexity. In short, our survey tool was not sufficiently EMR-like to serve as a substitute in our study. During the post-OSCE debriefing, participants explained that the survey-tool environment did not predispose them to utilizing the shortcuts they employed when using the EMR. The participants felt like they were taking a standardized examination (indeed, they were in an OSCE setting) and not at all as if they were taking care of patients using an EMR.

Furthermore, during debriefing sessions with the OSCE participants, we learned that the participants were keenly aware that the primary investigator and author (1) had made anti-copying/pasting statements in other venues and (2) that she was running this OSCE.

## Lessons learned

While determination and the ability to compromise are laudable personal attributes, as scientists we need to acknowledge when these attributes impede our ability to engage in good science. In every study design, there are essential components and considerations that are necessary to address the research question. In attempting to test a hypothesis, we had launched an investigation with the best possible scenario in mind (i.e., disabling the EMR copy & paste feature during real patient care activities and randomly assigning healthcare teams to one of two conditions). But this ideal design could not be realized. Determined to engage in our research, we began inserting compromises into our study design. Eventually, we had whittled away our study’s critical elements. What we were left with was a study design that could not test our hypothesis.

In this case, we may have been so eager to pursue our research interests (interests that we had been mulling over for years by that point!) and so pleased to have been granted some funding to support our research, that we did not question if the allocated resources and the reality of the situation at our institution would actually permit us to fully evaluate our hypothesis. In retrospect, we can identify at least four ‘non-negotiable’ elements we needed to have in order to study our hypothesis: (1) the use of the EMR; (2) the ability to disable the copy/paste function; (3) the ability to assess subject retention of general and patient-specific information; and (4) the ability to assess subject decision-making for specific scenarios presented in the OSCE. In hindsight, it is rather obvious that we could not study EMR-related behaviours in a context where the participants did not have access to an EMR. But, at the time, we were blinded by our passionate determination to study the copy/paste phenomenon. We were willing to engage in compromises to overcome contextual barriers. As a result, we failed to realize that the study design we were left with did not contain the essential components needed to be successful and to answer our research questions.

## Moral of the story

Scientists know that no study ever goes 100% according to plan. Therefore, we must be determined to see our studies through, and to overcome the obstacles that threaten the completion of our research. However, researchers are well advised to clearly articulate (if only to themselves) the essential components of their study design without which the project is unlikely to yield a useful answer to the study question. By knowing and documenting up-front the essential components of a study design, a researcher can evaluate when determination and compromise are inhibiting their efforts.
